# Epidemiological and Molecular Characterization of Invasive Meningococcal Disease in Italy, 2008/09-2012/13

**DOI:** 10.1371/journal.pone.0139376

**Published:** 2015-10-07

**Authors:** Arianna Neri, Patrizio Pezzotti, Cecilia Fazio, Paola Vacca, Fortunato Paolo D’Ancona, Maria Grazia Caporali, Paola Stefanelli

**Affiliations:** 1 Department of Infectious, Parasitic & Immuno-mediated Diseases, Istituto Superiore di Sanità, Rome, Italy; 2 National Center for Epidemiology, Surveillance and Health Promotion, Istituto Superiore di Sanità, Roma, Italy; Naval Research Laboratory, UNITED STATES

## Abstract

**Background:**

Following the introduction of meningococcal serogroup C conjugate vaccine in Italy in 2005, changes in the epidemiology of Invasive Meningococcal Disease (IMD) were expected. The study aims were to describe the epidemiological trend and to characterize the isolates collected during the period 2008/09-2012/13 by multilocus sequence typing (MLST). Data on laboratory confirmed meningococcal diseases from National Surveillance System of IMD were reported.

**Methods:**

Poisson regression models were used to estimate the incidence rate over time. Serogrouping and MLST were performed following published methods.

**Results:**

The incidence rate of laboratory confirmed meningococcal disease decreased from 0.33 per 100,000 population in 2008/09 to 0.25 per 100,000 population in 2012/13. The serogroup B incidence rate was significantly higher (p<0.01) than that of other serogroups, among all age groups. The significant decrease of the IMD incidence rate (p = 0.01) reflects the decrease of serogroup B and C, in particular among individuals aged 15–24 years old (p<0.01). On the other hand, serogroup Y incidence increased during the period (from 0.01/100,000 in 2008/09 to 0.02/100,000 in 2012/13, p = 0.05). Molecular characterizations revealed that ST–41/44 cc and ST–11 cc were the main clonal complexes identified among serogroup B and C isolates, respectively. In particular, ST–41/44 cc was predominant in all age groups, whereas ST–11 cc was not identified in infants less than 1 year of age.

**Conclusions:**

IMD incidence declined in Italy and serogroup B caused most of the IMD cases, with infants having the highest risk of disease. Continued surveillance is needed to provide information concerning further changes in circulating meningococci with special regard to serogroup distribution. Moreover, knowledge of meningococcal genotypes is essential to detect hyper-invasive strains.

## Introduction

Invasive Meningococcal Disease (IMD) remains an important public health concern worldwide, for its global epidemiology and the burden of IMD in different countries [1, 2]. IMD may be severe, often disabling, and sometimes fatal, especially among children, [3]. Although IMD occurs sporadically and outbreaks are rare, in some regions of the “African meningitis belt” devastating epidemic waves have been reported, [4].

The majority of *Neisseria meningitidis* strains which cause the invasive disease in Europe belongs mainly to serogroups B and C [5]. Of note, the groups at risk for IMD are patients less than 5 years of age, adolescents and young adults, [6].

Following the implementation of the meningococcal serogroup C conjugate (MCC) vaccination in many European countries, a decline in meningococcal serogroup C disease incidence has been observed, even if this serogroup is still circulating and is even responsible of small outbreaks, [7, 8, 9,10].

The MCC vaccine in Italy, introduced in 2005, is currently recommended in the National Immunization Plan (NIP 2012–2014), following regional policies, to all children from the 13^th^ to the 15^th^ month of age, and to 11–18 years old individuals, if not previously vaccinated, [11]. Data relating to MCC vaccine coverage was already described and estimated around 36.9% at the 12^th^ - 24^th^ months for the cohort 2006 and around 72% at 24^th^ months for the cohort 2009, [12, 13].

In Italy, in a previous analysis IMD due to serogroup C meningococci affected mostly children less than 4 years of age and adolescents, [14], but now the serogroup B is highly predominant, [15].

With regard to the licensure of the multicomponent vaccine against serogroup B vaccine (Bexsero®) in Europe [16], it is recommended following regional immunization strategies throughout the country.

Since several reports evidenced the antigenic and genetic diversity among *N*. *meningitidis* isolates, it is important to identify the molecular characteristics of the circulating meningococci [17].

In Italy, the National Surveillance System of IMD, as part of the National Surveillance System for Invasive Bacterial Diseases, and its European networking (European Centre for Disease Prevention and Control, ECDC), is a long-standing surveillance through the country.

In this study, we used the information derived from the National Surveillance System with the following aims: a) to describe IMD cases occurring in Italy from 2008/09 to 2012/13; b) to describe the clonal complexes of the circulating disease-associated meningococci.

## Methods

### Data sources

In Italy, all cases of IMD is based on mandatory notification. Information regarding age, sex, clinical picture, vaccination status, outcome of the disease, municipality of residence, nationality, municipality where the case occurred is routinely collected and managed using a dedicated database. Clinical isolates or samples are collected and stored at -80°C at the National Reference Laboratory (NRL) of the Istituto Superiore di Sanità (ISS).

### Bacterial strains and serogrouping

Meningococci are cultured on Thayer Martin agar plate with IsoVitalex 2% (Oxoid, Italy) and incubated in 5% CO_2_ atmosphere at 37°C for 18h. Serogroup identification, performed at local level, is confirmed by the NRL; moreover, the NRL performs serogroup identification when needed. Serogroup identification is carried out by slide agglutination with commercial antisera (Remel Europe Ltd, UK) or by PCR testing [18].

### DNA Extraction and MLST

Genomic DNA was extracted by QIAamp DNA minikit (Qiagen, Germany), according to the manufacturer’s protocol for Gram-negative bacteria.

Detection of DNA of *N*. *meningitidis* from sterile body site (cerebrospinal fluid or blood) was performed by real-time TaqMan PCR assay commercial kit (BIOSENSE, Italy).

MLST was performed following the guidelines included in the Neisseria pubMLST website to identify sequence type and clonal complex (cc) (http://neisseria.org/neisseria/) [17].

### Ethical Statement

Ethical approval for the study was not required, because all the examined cases were managed as part of routine diagnostics (standard care). Patient’s data were recorded anonymously in separated files under the umbrella of the national surveillance system.

### Statistical analysis

Incidence rates were calculated using as denominator the Italian population size provided in the web-site (www.demo.istat.it) of the National Bureau of Census. These numbers are available per calendar year, sex, age, region, and municipality. Incidence rates were calculated stratifying cases by epidemiological year, age class (i.e., <1, 1–4, 5–14, 15–24, ≥25 years old), serogroup and regions with low (<20%) (n = 8) and high (≥20%) (n = 12) percentage of unknown (UNK) serogroup [19].

Poisson regression models were used to evaluate the temporal trend of the incidence rates (both overall, and stratified by regions with low and high percentage of UNK serogroup, and by age group).

Statistical analysis was performed using STATA 12.0 (StataCorp. 2011. Stata Statistical Software: Release 12. College Station, TX: StataCorp LP).

## Results

### Invasive Meningococcal Disease

Out of 794 laboratory confirmed cases of meningococcal disease in Italy, from 2008/09 to 2012/13 epidemiological years, 410 meningococcal isolates and 31 positive clinical samples were sent to the NRL.

The clinical presentation of IMD was reported for 100% of cases. As expected, meningitis and septicaemia represented the main clinical syndromes: in particular, meningitis was reported in 55% (436/794) of the cases, sepsis in 29% (230/794) and meningitis and sepsis in 15.3% (122/794). Sepsis was more frequently associated with serogroup C (32.5%; 57/162) compared to serogroup B (29.8%; 107/359) and Y (25.8%; 16/62); however, there was no statistically significant association between clinical manifestation and serogroup (p = 0.07), (S1 Table).

Laboratory diagnosis was known for 97.8% (777/794) of cases and was confirmed in 66.5% (517/777) by culture, in 14% (107/777) by PCR, in 7.5% (58/777) by both culture and PCR, and in 12% (95/777) by microscopy or antigen test.

The overall incidence of laboratory confirmed meningococcal disease during the period was 0.26 per 100,000 inhabitants (Table 1).

**Table 1:** Number of cases and incidence of meningococcal disease in Italy by epidemiological year.

		2008/09		2009/10		2010/11		2011/12		2012/13		Total	
**Italy**		**N**	**%**	**N**	**%**	**N**	**%**	**N**	**%**	**N**	**%**	**N**	**%**
	**Known serogroup**	170	85.4	109	75.2	121	78.6	107	72.8	102	68.5	609	76.7
	**Unknown serogroup**	29	14.6	36	24.8	33	21.4	40	27.2	47	31.5	185	23.3
	**Total cases**	199		145		154		147		149		794	
	**Population**	59832179		60192698		60483385		60010325		60010325		300528911	
	**Incidence**	0.33		0.24		0.25		0.24		0.25		0.26	
**Regions with <20% of cases with unknown serogroup**		**N**	**%**	**N**	**%**	**N**	**%**	**N**	**%**	**N**	**%**	**N**	**%**
	**Known serogroup**	123	93.9	79	82.3	69	87.3	76	85.4	80	83.3	427	87.0
	**Unknown serogroup**	8	6.1	17	17.7	10	12.7	13	14.6	16	16.7	64	13.0
	**Total cases**	131		96		79		89		96		491	
	**Population**	26901733		27131868		27317071		27118025		27118025		135586720	
	**Incidence**	0.49		0.35		0.29		0.33		0.35		0.36	
**Regions with ≥20% of cases with unknown serogroup**		**N**	**%**	**N**	**%**	**N**	**%**	**N**	**%**	**N**	**%**	**N**	**%**
	**Known serogroup**	47	69.1	30	61.2	52	69.3	31	53.4	22	41.5	182	60.1
	**Unknown serogroup**	21	30.9	19	38.8	23	30.7	27	46.6	31	58.5	121	39.9
	**Total cases**	68		49		75		58		53		303	
	**Population**	32930447		33060830		33166315		32892300		32892300		164942191	
	**Incidence**	0.21		0.15		0.23		0.18		0.16		0.18	

The serogroup was unknown in 185 cases (185/794; 23%) out of the total (Table 1). For this reason, the analysis regarding the serogroups was performed on the overall country and on the Italian Regions divided in two groups: regions with <20% and regions with ≥20% of annual cases with UNK serogroup. A total of 491 IMD cases occurred in regions <20% of annual cases with UNK serogroup, and 303 cases in regions with a rate of annual cases with UNK serogroup ≥20% (Table 1).

The total incidence of laboratory confirmed meningococcal disease significantly declined from 0.33 per 100,000 inhabitants in 2008/09 to 0.25 per 100,000 inhabitants in 2012/13 (p = 0.01), (S1 Fig).

A similar trend was observed in regions with <20% of annual cases with UNK serogroup (p = 0.02) (S1 Fig). No significant changes in the incidence in the regions with ≥20% of annual cases with UNK serogroup was observed (p = 0.40), (data not shown).

Over the five years, serogroup B cases occurred most frequently (0.12 per 100,000), followed by serogroup C (0.05 per 100,000), serogroup Y (0.02 per 100,000), and other serogroups (including A, *cnl*, E, W, X) (0.01 per 100,000), (Table 2).

**Table 2:** Number and incidence of IMD cases by serogroup and age groups.

		**B**		**C**		**Y**		**Other** ^a^		**UNK**		**Total**	
**Italy**	**Age group**	**N**	**rate**	**N**	**rate**	**N**	**rate**	**N**	**rate**	**N**	**rate**	**N**	**rate**
	**<1**	58	2.09	12	0.43	2	0.07	2	0.07	21	0.76	95	3.42
	**1–4**	81	0.71	28	0.25	4	0.04	3	0.03	24	0.21	140	1.24
	**5–14**	63	0.22	17	0.06	21	0.07	3	0.01	32	0.11	136	0.48
	**15–24**	64	0.21	34	0.11	9	0.03	3	0.01	32	0.11	142	0.47
	**>25**	93	0.04	71	0.03	26	0.01	15	0.01	76	0.03	281	0.12
	**Total**	359	0.12	162	0.05	62	0.02	26	0.01	185	0.06	794	0.26
		**B**		**C**		**Y**		**Other** ^a^		**UNK**		**Total**	
**Regions with <20% of cases with unknown serogroup**	**Age group**	**N**	**rate**	**N**	**rate**	**N**	**rate**	**N**	**rate**	**N**	**rate**	**N**	**rate**
	**<1**	36	2.87	5	0.40	0	0.00	1	0.08	8	0.64	50	3.98
	**1–4**	59	1.16	17	0.34	1	0.02	2	0.04	5	0.10	84	1.66
	**5–14**	50	0.42	10	0.08	15	0.13	0	0.00	10	0.08	85	0.71
	**15–24**	48	0.40	25	0.21	3	0.02	2	0.02	16	0.13	94	0.78
	**>25**	74	0.07	51	0.05	15	0.01	13	0.01	25	0.02	178	0.17
	**Total**	267	0.20	108	0.08	34	0.03	18	0.01	64	0.05	491	0.36

Other^a^ serogroup: A, *cnl*, E, W and X

UNK, cases with unknown serogroup

A decrease of incidence rates for serogroup B (from 0.16 in 2008/09 to 0.09 in 2012/13 per 100,000; p<0.01) and for serogroup C (from 0.10 in 2008/09 to 0.05 in in 2012/13 per 100,000; p<0.01) was also observed. However, when excluding the epidemiological year 2008/09, serogroup C showed a stable trend (from 0.03 in 2009/10 to 0.05 in 2012/13 per 100,000, p = 0.17).

The incidence rate of serogroup Y showed a significant increase during the study period (from 0.01 in 2008/09 to 0.02 in 2012/13 per 100,000; p = 0.05).

Infants under 1 year of age (3.42/100,000) and children aged 1 to 4 years, (1.24/100,000) were the most affected. The lowest of the total IMD incidence rate was observed in adults ≥ 25 years (0.12/100,000), (Table 2).

Serogroup B incidence rate was significantly more common than all other serogroups, in all age groups (p<0.01) although a low difference in terms of incidence rate in the population aged ≥ 25 years was found. Serogroup C incidence rate decreased with the increase of the age. Serogroup Y incidence rate was higher among infants>1 year and children aged 5–9 years (0.07/100,000), Table 2.

A similar age distribution of IMD cases was observed in regions with <20% of annual cases with UNK serogroup, (Table 2).

**Fig 1:**
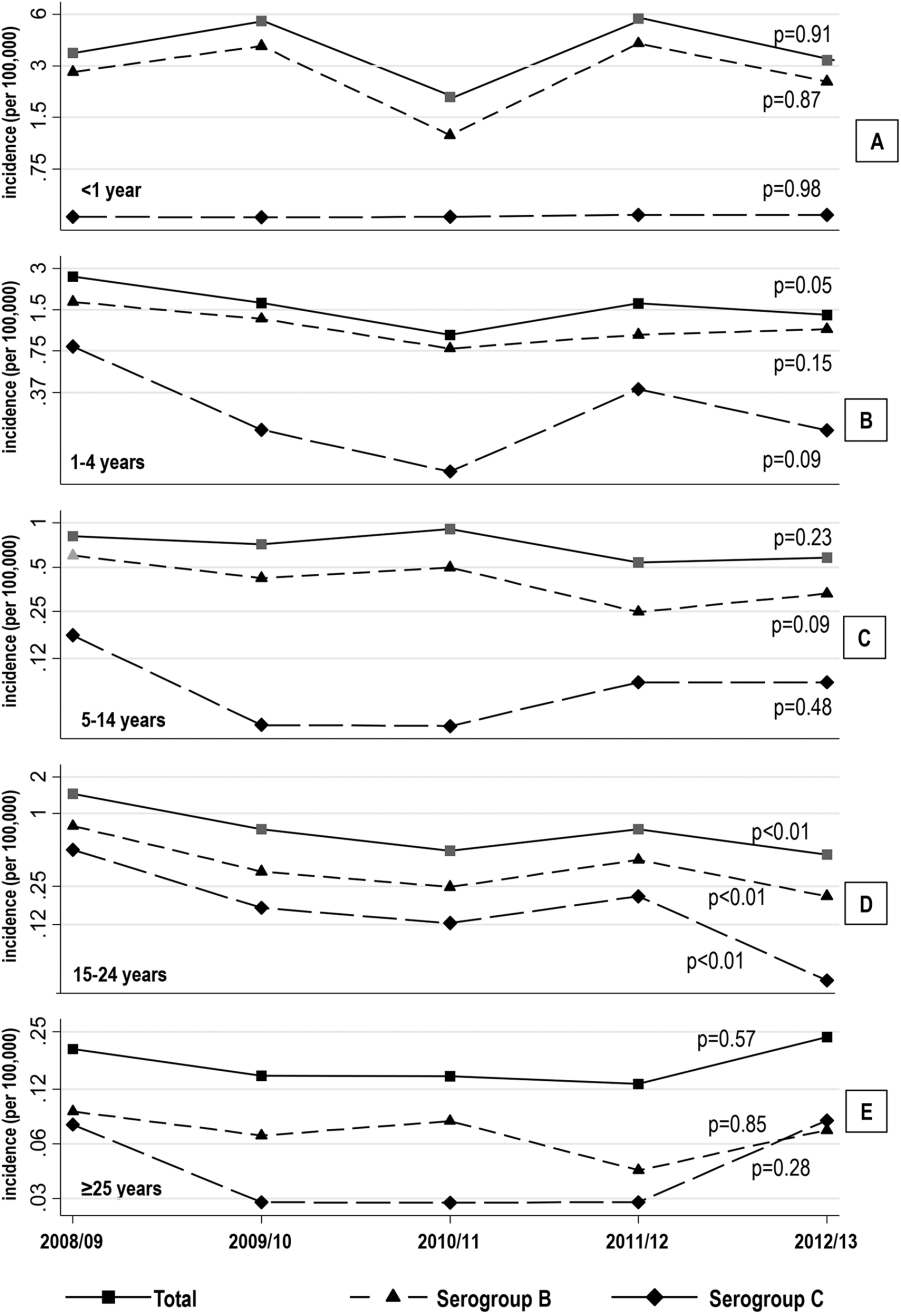
Annual incidence, on logarithmic scale, of IMD in Italian Regions with <20% of annual cases with UNK serogroup by age group: A) <1, B) 1-to–4, C) 5-to–14, D) 15-to–24, E) ≥25 years. *p*-values from Poisson univariate regression models with epidemiological year as covariate are in the right side.

Fig 1 shows the temporal trend of the incidence rate for all serogroups combined and for the serogroup B and C, separately. For the age group <1 year, no significant changes in the incidence rates of all serogroups combined as well as for the serogroup B and C, respectively were observed (Fig 1A). For the age group 1–4, there was a significant decline for all serogroups combined; a similar decrease was observed both for serogroup B and C, respectively, but this decline was not statistically significant (Fig 1B). Regarding the other three age groups (Fig 1C, 1D and 1E), only for age group 15–24 years (Fig 1D) a statistically significant decline for the overall incidence rate, as well as, for the serogroup B and C, respectively, was observed.

### Molecular investigation

A total of 21 known clonal complexes (cc_s_) were identified. The most commonly detected were ST–41/44 cc (76/344, 22%), ST–11 cc (66/344, 19%), and ST–23 cc (44/344, 13%).

Among serogroup B, 13 cc_s_ were identified and the most common were ST–41/44 cc (73/182, 40%), ST–32 cc (22/182, 12%), ST–162 cc (22/182, 12%) and ST–269 cc (14/182, 8%).

Among serogroup C, 8 cc_s_ were identified and the most common were: ST–11 cc (62/101, 61%), and ST–334 cc (19/101, 19%).

The majority of cases due to serogroup Y belonged to ST–23 cc (43/45, 96%).

Four cc_s_ exclusively found among serogroup B were: ST- 162 cc, ST–865 cc, ST–213 cc and ST–461 cc. The ST–334 and ST–8 cc were restricted to serogroup C.

A total of 23 isolates (23/344; 6.7%), belonging to sequence types not currently assigned to any clonal complex, were classified as unknown (UNK).

**Fig 2:**
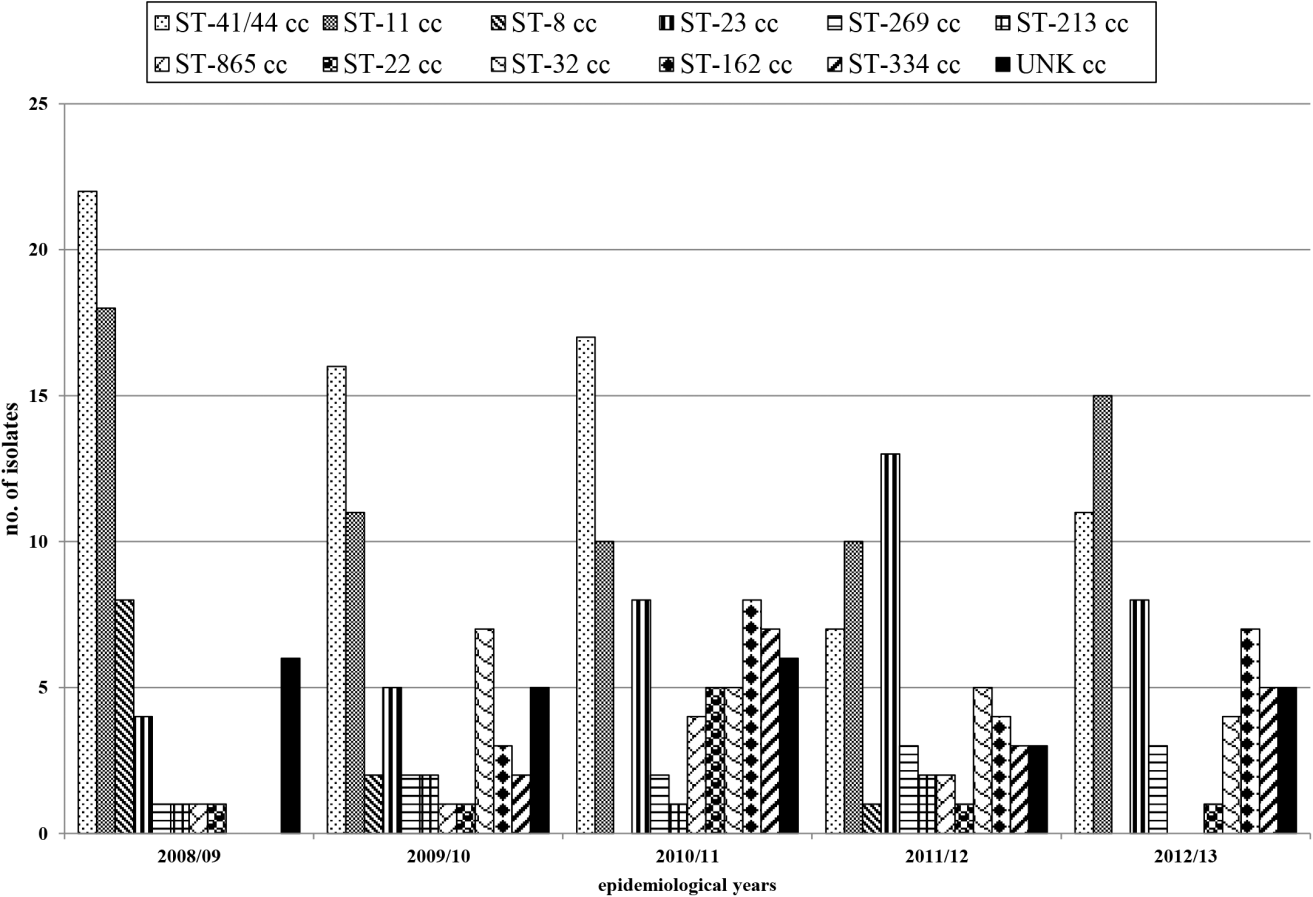
Distribution of the main clonal complexes (cc_s_) identified among meningococci collected in the study period.

The ST–41/44 cc and the ST–11 cc tended to decrease from 2008/09 to 2012/13. The ST–8 cc decreased and disappeared from 2008/09 to 2012/13, with 1 ST–8 cc strain isolated in 2011/12. The ST–23 cc increased from 2008/09 to 2012/13, with a peak in 2011/12. Since 2009/10, ST–32 cc, ST–162 cc, and ST–334 cc, were identified (Fig 2).

Among infants <1 year and among children 1–4 years, ST–41/44 cc was the prevalent; ST–23 cc and ST–41/44 cc were also the prevalent in subjects from 5 to 14 years; in subjects from 15 to 24 years, ST–41/44 cc and ST–11 cc were both the main cc_s_. Moreover, the majority of ST–32 cc isolates were found among individuals aged from 15 to 24years. In adults ≥25 years, ST–11 cc and ST–23 cc were the most frequently identified (Table 3).

**Table 3:** Age distribution of the most common clonal complexes identified in Italy, from 2008/09 to 2012/13.

	Age group (number per serogroup)					
Clonal complex	<1	1–4	5–14	15–24	>25	Total
**ST–41/44**	13(12B, 1C)	14 (14B)	15 (14B, 1C)	17 (17B)	17 (16B, 1C)	76
**ST–11**	0	9 (9C)	6 (6C)	15 (14C, 1B)	36 (33C, 2B, 1W)	66
**ST–8**	3 (3C)	3 (3C)	2 (2C)	3 (3C)	0	11
**ST–334**	4 (4C)	3 (3C)	2 (2C)	2 (2C)	8 (8C)	19
**ST–23**	0	2 (2Y)	18 (18Y)	3 (3Y)	21 (20Y, 1C)	44
**ST–32**	3 (3B)	5 (5B)	3 (3B)	9 (8B, 1C)	2 (2B)	22
**ST–162**	4 (4B)	4 (4B)	4 (4B)	1 (1B)	9 (9B)	22
**ST–269**	3 (3B)	4 (4B)	3 (3B)	1 (1C)	4 (4B)	15

## Discussion

Invasive meningococcal disease (IMD) is a serious illness that despite an early intervention and modern intensive care can become fatal in few hours. Vaccination is the only strategy to prevent the disease and to improve the herd protection in the population, thus reducing morbidity and mortality. According to IMD surveillance data reported by ECDC, the incidence of confirmed cases in 28 European countries continues to decrease: from 0.95 per 100,000 in 2008 to 0.68 per 100,000 in 2012 [5].The main reason for this decline could be the introduction of MCC vaccination in several countries [20]. Moreover, the effect of vaccination on carriage status results in a long-term reduction of the disease [21].

In Italy, IMD incidence rate showed cyclical fluctuations, in particular regarding to the total number of cases due to serogroup B and C, respectively [22, 14]. Moreover, other factors such as climate and geographical variation have been associated to a gradient in the IMD incidence in the country [23].

The risk of meningococcal disease clearly varies with age and serogroup. Meningococcal carriage is most common in teenagers whereas the invasive disease is reported mainly in infants and in a secondary peak observed in teenagers [24].

The serogroup distribution observed in this study was similar to that reported in most European countries, with serogroup B and C responsible for the majority of disease, followed by serogroup Y [5]. Serogroup B was responsible for the majority of IMD cases in all age groups, with a statistically significant higher incidence compared to any other serogroup. As already reported in Italy, serogroup B is the predominant among infants [15]. The Bexsero has been recently introduced in Italy even if with differences among the Regions. In a previous study [25], the potential Bexsero strain coverage has been estimated at 87% (95% CI 70–93).

In comparison to other serogroups, the number of IMD cases due to serogroups A and W is rare, in Italy. Whereas, an increase of IMD due to serogroup W have been reported in England and Wales since 2009 due to the emergence and clonal expansion of a single clone [26]. Serogroup Y increased as already reported in other European countries and worldwide [27, 28, 29]. These results may contribute in the revision of catch-up or booster vaccination, hopefully with the quadrivalent conjugate vaccine ACWY. The vaccine is available and recommended in Italy for at risk groups and for people who live or travel to countries where meningococcal disease is hyperendemic or epidemic. Moreover, it can be administered to children from 12 months of aged who have not received MCC vaccine or to adolescents aged between 12 and 16 years, as booster of MCC vaccine and for a complete coverage.

In the present analysis, serogroup B meningococci resulted more heterogeneous than serogroup C. In particular, the majority of clonal complexes among serogroup B were: ST–41/44 cc, ST–32 cc, ST–162 cc and ST–269 cc. These clonal complexes were distributed in all age groups.

The clonal complexes among serogroup C meningococci isolated from 2008/09 to 2012/13 were: ST–11 cc, ST–334 cc and ST–8 cc. Serogroup C ST–8 cc, mainly in persons aged less than 25 years, decreased and the serogroup C ST–334 cc, identified in adults, still increased. The majority of serogroup C ST–11 cc isolates were identified in all age groups except for infants less than 1 year of age, as already described [30].

Previously, serogroup C IMD cases in Italy were mainly associated with ST–8 cc (2003–2005) [31] and ST-11cc (2007–2008) [32].

As with any national surveillance study, the estimation is likely to represent a possible underreporting of disease incidence. Moreover, the relatively high proportion of cases with UNK serogroup (23.3%) could affect the data. Nevertheless, results from Regions with a low rate of UNK serogroup in our dataset are consistent with those collected in the rest of the country.

In conclusion, data from the national surveillance system provides information on IMD in the country up to 2013, before the introduction of meningococcal B vaccination in the country. Molecular typing analysis permits to monitor the changes in the distribution of those hyper-virulent clonal complexes recognized as responsible of outbreaks or rapid endemic expansion, demanding a continuous surveillance for the possible genetic pressure due to the immunization policies.

## Supporting Information

S1 Table Clinical pictures and serogroups of IMD cases from 2008/09 to 2012/13, Italy. (DOCX)

S1 Fig Annual incidence, on logarithmic scale, of IMD cases in Italy (A) and in Regions with <20% of IMD cases with UNK serogroup (B). (TIF)
