# Supplementary material for: Epidemiological and Molecular Characterization of Invasive Meningococcal Disease in Italy, 2008/09-2012/13
Source: PLoS One. 2015 Oct 7;10(10):e0139376. doi: 10.1371/journal.pone.0139376 (PMC4596568; doi:10.1371/journal.pone.0139376)
Supplement: S1 Table — (DOCX) [file pone.0139376.s001.docx]

|  | **B** | | **C** | | **Y** | | **Other^a^** | | **UNK^b^** | | **Total** |
| --- | --- | --- | --- | --- | --- | --- | --- | --- | --- | --- | --- |
| **Clinical picture** | **N** | **%** | **N** | **%** | **N** | **%** | **N** | **%** | **N** | **%** | **N** |
| **Meningitis** | 185 | 51.5 | 78 | 48.1 | 37 | 59.7 | 15 | 57.7 | 121 | 65.4 | 436 |
| **Saepticemia** | 107 | 29.8 | 57 | 35.2 | 16 | 25.8 | 10 | 38.5 | 40 | 21.6 | 230 |
| **Meningitis/Saepticemia** | 63 | 17.5 | 26 | 16.0 | 8 | 12.9 | 1 | 3.8 | 24 | 13.0 | 122 |
| **Total** | 359 |  | 162 |  | 62 |  | 26 |  | 185 |  | 794 |

*^a^* A, *cnl,* E,W,X

*^b^* unknown serogroup
