# Supplementary figures and images for: Epidemiological and Molecular Characterization of Invasive Meningococcal Disease in Italy, 2008/09-2012/13
Source: PLoS One. 2015 Oct 7;10(10):e0139376. doi: 10.1371/journal.pone.0139376 (PMC4596568; doi:10.1371/journal.pone.0139376)

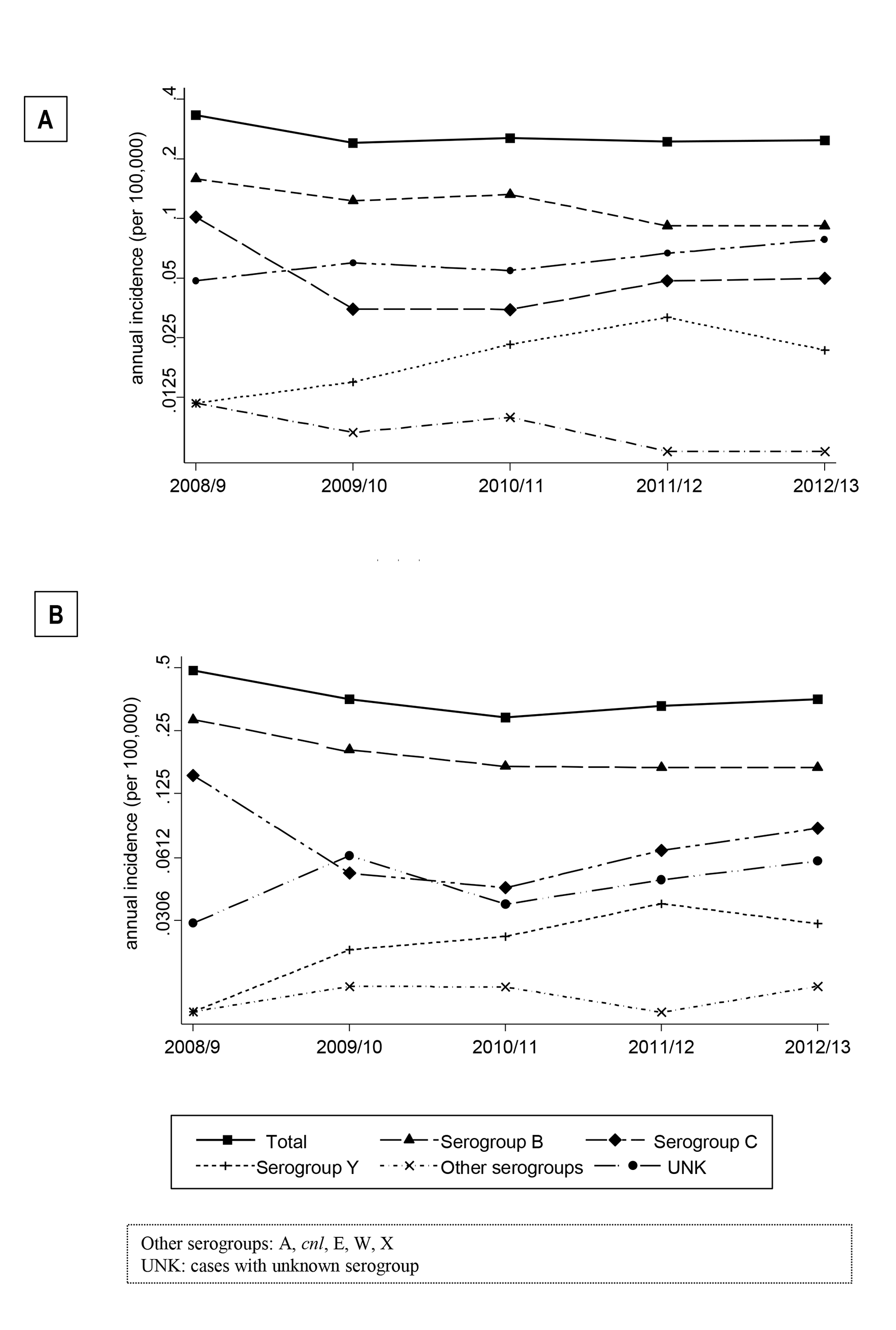

Supplement: S1 Fig — (TIF) [file pone.0139376.s002.tif]
